# Comparison of laryngeal tube evo with tracheal intubation during intra-arrest-ventilation using biphasic positive pressure ventilation - a prospective randomized controlled body donor study

**DOI:** 10.1186/s13054-026-05902-4

**Published:** 2026-03-28

**Authors:** Gerrit Jansen, Lydia Johnson Kolaparambil Varghese, Beate Brand-Saberi, Cornelius Christopher Falk, Maximilian Feth, Eckart Förster, Tamar Gelashvili, Harald Genzwürker, Annika Hoyer, Marcel Ihlenfeld, Jan-Steffen Pooth, Amy Thomasseck, Justin Trenkel, Jens Tiesmeier

**Affiliations:** 1 University Department of Anaesthesiology, Intensive Care Medicine, Emergency Medicine and Pain Medicine, Ruhr University of Bochum, Johannes Wesling Klinikum Minden, Hans- Nolte-Straße 1, 32429 Minden, Germany; 2 Institute of Anatomy, Medical Faculty of Ruhr University Bochum, Ruhr University Bochum, Universitätsstraße 150, 44801 Bochum, Germany; 3 Medical Faculty of Ruhr University Bochum, Ruhr University Bochum, Universitätsstraße 150, 44801 Bochum, Germany; 4 Dpt. of Anesthesiology, Critical Care, Emergency and Pain Medicine, German Armed Forces Hospital Ulm, 89081 Ulm, Germany; 5 Medical Faculty, University of Heidelberg, Heidelberg, Germany; 6 Medical School OWL, Biostatistics and Medical Biometry, Bielefeld University, Universitätsstraße 25, 33615 Bielefeld, Germany; 7 Medical Faculty, University Emergency Department. Medical Center Freiburg, University of Freiburg, Hugstetter Straße 55, 79106 Freiburg, Germany; 8 Institute for Anaesthesiology, Intensive Care- and Emergency Medicine, MKK-Hospital Luebbecke, Ruhr-University Bochum, Campus OWL, Germany

**Keywords:** Resuscitation, Airway management, Ventilation, Artificial, Advanced cardiac life support

## Abstract

**Background:**

This study evaluates the effects of the new supraglottic airway-device laryngeal tube evo (LT^®^evo) versus that of tracheal intubation (TI) concerning the target parameters of ventilation therapy during mechanical chest compressions in intra-arrest-ventilation with biphasic-positive-airway-pressure-ventilation.

**Methods:**

This prospective randomized crossover study involving Thiel-embalmed human body donors, compares TI with LT^®^evo using biphasic-positive-airway-pressure-ventilation during mechanical cardiopulmonary resuscitation. Each body donor underwent ventilation with both airway-devices in randomized order. Ventilatory parameters were recorded at ventilator flow sensors. Primary endpoint was expiratory tidal volume (VT_e_), secondary endpoints included expiratory tidal volume per kg ideal body weight (VT_e_/kgIBW); delta tidal volume (ideal-VT_e_; ΔVT); leakage volume (V_Leak_); peak (P_Peak_), mean (P_Mean_), plateau (P_Plat_) pressures; and respiratory rates.

**Results:**

Five body donors were included. Linear regression analyses with random intercepts revealed lower VT_e_ (-295.7 ± 8 ml; 95%CI:-339.2 to -252.2); *p* < 0.0001), VT_e_/kgIBW (-5.0 ml/kg; 95%:-5.7 to -4.2; *p* < 0.0001), P_Peak_ (-19.0mbar; 95%CI:-23.1 to -14.9; *p* < 0.0001) and P_Mean_ (-2mbar; 95%CI:-3.2 to -0.8 ;*p* = 0.001) but higher ∆VT (296.0 ml; 95%CI:252.5 to 339.4; *p* < 0.0001) and V_Leak_ (52.7%; 95%CI:41.6 to 63.7; *p* < 0.0001) for LT^®^evo. While all tracheal-intubated body donors (100%) were successfully ventilated with biphasic-positive-airway-pressure-ventilation (VT_e_/kgIBW > 2 ml/kg), only one of five body donors (20%) could be ventilated via LT^®^evo.

**Conclusion:**

In this body donor model of biphasic-positive-airway-pressure-ventilation during mechanical cardiopulmonary resuscitation, TI provided higher VT_e_ and lower leakage than LT^®^evo did. LT^®^evo ventilation frequently failed to achieve tidal volumes above the estimated dead space. These findings suggest that LT^®^evo may be poorly suited for intra-arrest ventilation using biphasic-positive-airway-pressure-ventilation during mechanical cardiopulmonary resuscitation. When the LT^®^evo is used in this setting, a synchronous 30:2 ventilation strategy may represent a safer alternative.

**Trial registration:**

German Clinical Trials Register; unique identifier: DRKS00037836; registration date: September 09, 2025.

**Supplementary Information:**

The online version contains supplementary material available at 10.1186/s13054-026-05902-4.

## Background

The optimal management of the airway in out-of-hospital cardiac arrest has been a subject of debate for decades [1–4]. Comparative studies on the effects of tracheal intubation (TI) and various supraglottic airway (SGA) devices have shown somewhat contradictory results. While TI is usually performed by emergency medical personnel because of its complexity, SGA devices are particularly established by nonmedical emergency service personnel and are widely used internationally [5, 6].

Recent guideline updates have refined these recommendations. The 2025 European Resuscitation Council guidelines recommend the use of an i-Gel^®^-laryngeal mask over a laryngeal tube because of favourable success and lower complication rates. Laryngeal tube evo (LT^®^evo) is a new and innovative laryngeal tube whose effects have not yet been evaluated in the context of mechanical intra-arrest ventilation during resuscitation [7]. For the first time, the current European Resuscitation Council resuscitation guidelines provide specific recommendations on the use of volume-controlled or pressure-regulated ventilation modes in the event of mechanical intra-arrest ventilation [7]. Although volume-controlled ventilation modes are widely used in emergency care and have been associated with favourable prognostic outcomes [8], studies in tracheally intubated patients suggest that pressure-controlled modes, including biphasic-positive-airway-pressure-ventilation, provide more consistent control of delivered tidal volumes [9]. However, no studies have investigated the effects of LT^®^evo when biphasic-positive-airway-pressure-ventilation is used.

This prospective, randomized crossover body donor study was therefore designed to compare TI with LT^®^evo during asynchronous intra-arrest ventilation using biphasic-positive-airway-pressure-ventilation under continuous mechanical chest compressions. The primary aim was to evaluate differences in expiratory tidal volume, with secondary outcomes examining leak, airway pressures, and other key ventilation metrics.

## Methods

### Study design

This prospective, randomized crossover study was conducted in October 2025 at the Institute of Anatomy of Ruhr University Bochum in collaboration with the Clinical Anatomical Research and Training Centre. The study protocol was approved by the ethics committee of Ruhr University Bochum, Ostwestfalen-Lippe, Bad Oeynhausen, Germany (Ref. 2025 − 1341_2). This trial was registered in the German Clinical Trials Register at DRKS00037836 (https://www.drks.de/DRKS00037836) on 9th September 2025 before data collection [10]. All procedures adhered to the principles of the Declaration of Helsinki [11]. The structure of the paper is based on the current CONSORT guidelines [12].

Because of the limited availability of Thiel-embalmed body donors, this study was part of a broader experimental program evaluating multiple ventilation strategies. However, the present analysis concerns only the biphasic-positive-airway-pressure-ventilation ventilation runs.

### Participants

Adult Thiel-embalmed human body donors were included [13]. Body donors with abnormal airways, tracheostomies, and premortem acute respiratory distress syndrome, and severe lung or thoracic injuries, such as undrained pneumothorax, or severe aspiration, were excluded.

The body donors donated their bodies to the Institute of Anatomy at Ruhr University Bochum after their death for scientific and study purposes. Sex and gender were recorded based on primary sexual characteristics. Race/ethnicity was not recorded.

### Randomisation and masking

Both the order of the airway devices and the ventilation modes were randomized per body donor using simple randomization generated by a computer-based method. Masking was not achieved. All participants received the same interventions. All Thiel-fixed body donors that could be provided by the Institute of Anatomy, under consideration the exclusion criteria, were included.

### General Preparation of the body donors

After an external postmortem examination with documentation of the biological sex, actual body weight, and size of the body donor, the ideal body weight was calculated using Broca’s formula [14]. This process was followed by tracheal intubation via Mallinckrodt tubes (male = 8.0 inner diameter (in mm), female = 7.0 inner diameter, cuff pressure = 40 cmH2O) and bronchoscopy to check the tube position and remove secretions.

In preparation for the experiment, the lungs were recruited for four minutes by pressure-controlled ventilation (inspiratory pressure (P_Insp_) = 35 mbar, positive end-expiratory pressure (PEEP) = 12 mbar, and respiratory rate = 10 min^− 1^) via MEDUMAT Standard^2^ (WEINMANN Emergency, Medical Technology GmbH + Co. KG, Hamburg, Germany) and extubated after recruitment. All procedures were performed by a specialist in anaesthesiology.

### Airway management

After lung recruitment, two airway devices were inserted according to the predefined randomization list: (1) TI (control group) and (2) LT^®^evo (VBM Medizintechnik GmbH, Sulz am Necker, Germany). The size of the respective LT^®^evo was selected in accordance with the manufacturer’s specifications. The time-to-airway was measured for each airway device and defined as the time between grasping and releasing the airway device. The cuff pressure was limited to 40 cmH_2_O for TI and LT^®^evo. Device placement was verified by bronchoscopy after insertion. Before each ventilation period, a standardized lung recruitment protocol involving five manual insufflations via the MEDUTrigger (MEDUMAT Standard^2^, WEINMANN Emergency, Medical Technology GmbH + Co. KG, Hamburg, Germany) was performed to minimize carry-over effects. After four minutes of intra-arrest ventilation, the airway device was removed, and the next airway device was inserted according to the randomization list and checked again as prevously described.

### Laryngeal tube evo

**Fig. 1:**
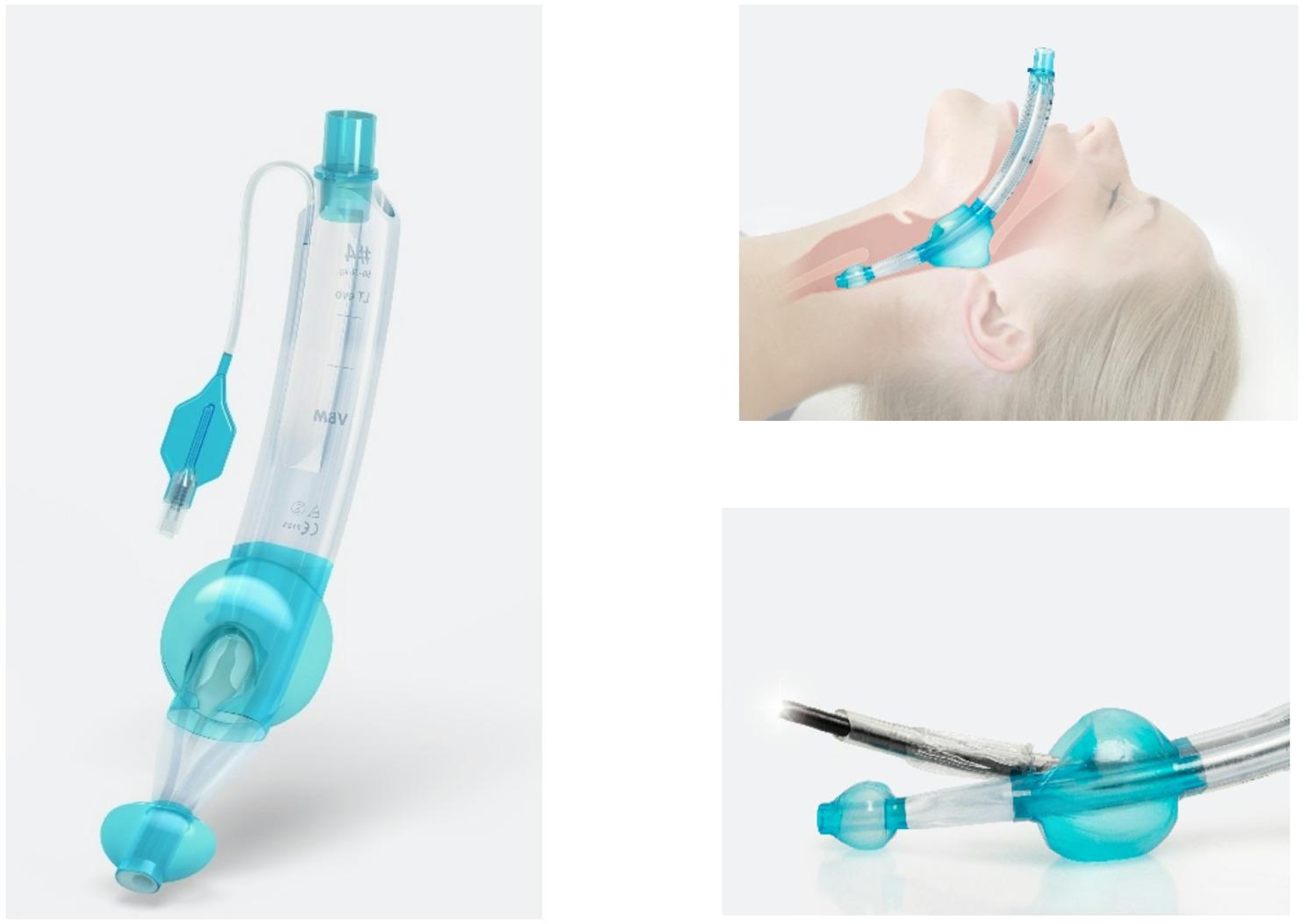
Laryngeal tube EVO

The second-generation SGA LT^®^evo represents a further development of the traditional laryngeal tube LTS-D (VBM Medizintechnik GmbH, Sulz am Necker, Germany) which has been marketed for years: It is made of more flexible and softer material than its predecessor and has a modified shape that adapts to the anatomical contours of the relaxed upper airways and is designed to make insertion easier; its design is intended to ensure easy handling. Like its predecessor, the LT^®^evo has a proximal cuff to seal the oropharyngeal area with modified cuff geometry to enhance seal pressure (< 60 cmH2O) and effective sealing, as well as a distal cuff to seal the oesophageal entrance; its widened ventilation lumen and an integrated ramp at the end of the ventilation lumen allow for fibreoptic-guided intubation through the device. An illustration of the LT^®^evo is shown in Fig. 1.

### Ventilation and chest compressions

Ventilation was performed using MEDUVENT (WEINMANN Emergency, Medical Technology GmbH + Co. KG, Hamburg, Germany) for four minutes with biphasic positive pressure ventilation (inspiratory pressure (P_Insp_) = 20 mbar, positive end-expiratory pressure (PEEP) = 5 mbar, and respiratory rate = 10/min).

Chest compressions were performed in a standardized manner using a mechanical chest compression device Corpuls-CPR (GS Elektromedizinische Geräte G. Stemple GmbH, Kaufering, Germany; frequency = 100/min; and depth = 5.5 cm) [7]. In the various resuscitation scenarios, sonographic exclusion of pneumothorax was performed, and if present, drainage was facilitated by inserting a chest drain in the Bülau position.

### Measurement of the ventilation parameters

During the experiments, the ventilation parameters were measured at the flow sensor and recorded via MEDUVENT. The following parameters were recorded continuously without prespecified measurement time points: expiratory tidal volume (VT_e_ in ml), inspiratory minute volume (MV_i_ in l), leakage volume (V_Leak_ in %), peak pressure (P_Peak_ in mbar), and mean pressure (P_Mean_ in mbar). The calculated parameters were the ideal tidal volume (VT_Ideal_=7 ml/kgIBW), ΔVT (in ml; difference between VT_Ideal_-VT_e_), and VT_e_/kgIBW (in ml/kgIBW). A detailed overview of the recorded and calculated ventilation parameters is provided in Additional material 1 (File name: Additional File; format: .pdf; title: Additional material 1; description: Table of measured ventilation parameters and calculation methods).

### Outcomes

The primary endpoint was VT_e_. The secondary endpoints included ΔVT, V_Leak_, VT_e_/kgIBW, P_Peak_, P_Mean_, and P_Plat_.

### Statistical analysis

Data were first analysed descriptively according to the variable type: means and standard deviations for continuous variables and numbers and proportions for categorical variables. For the primary outcome, a linear regression model with a random intercept was applied to account for potential intracardiac correlations. For all secondary outcomes, we again used linear regression models with random intercepts. As effect measures for all analyses, we report regression coefficients with 95% confidence intervals (95% CIs) and p-values if appropriate. A p-value ≤ 0.05 was considered to indicate statistical significance. All analyses were performed using the statistical software SAS 9.4^®^ (SAS Institute Inc., Cary, NC).

## Results

**Fig. 2:**
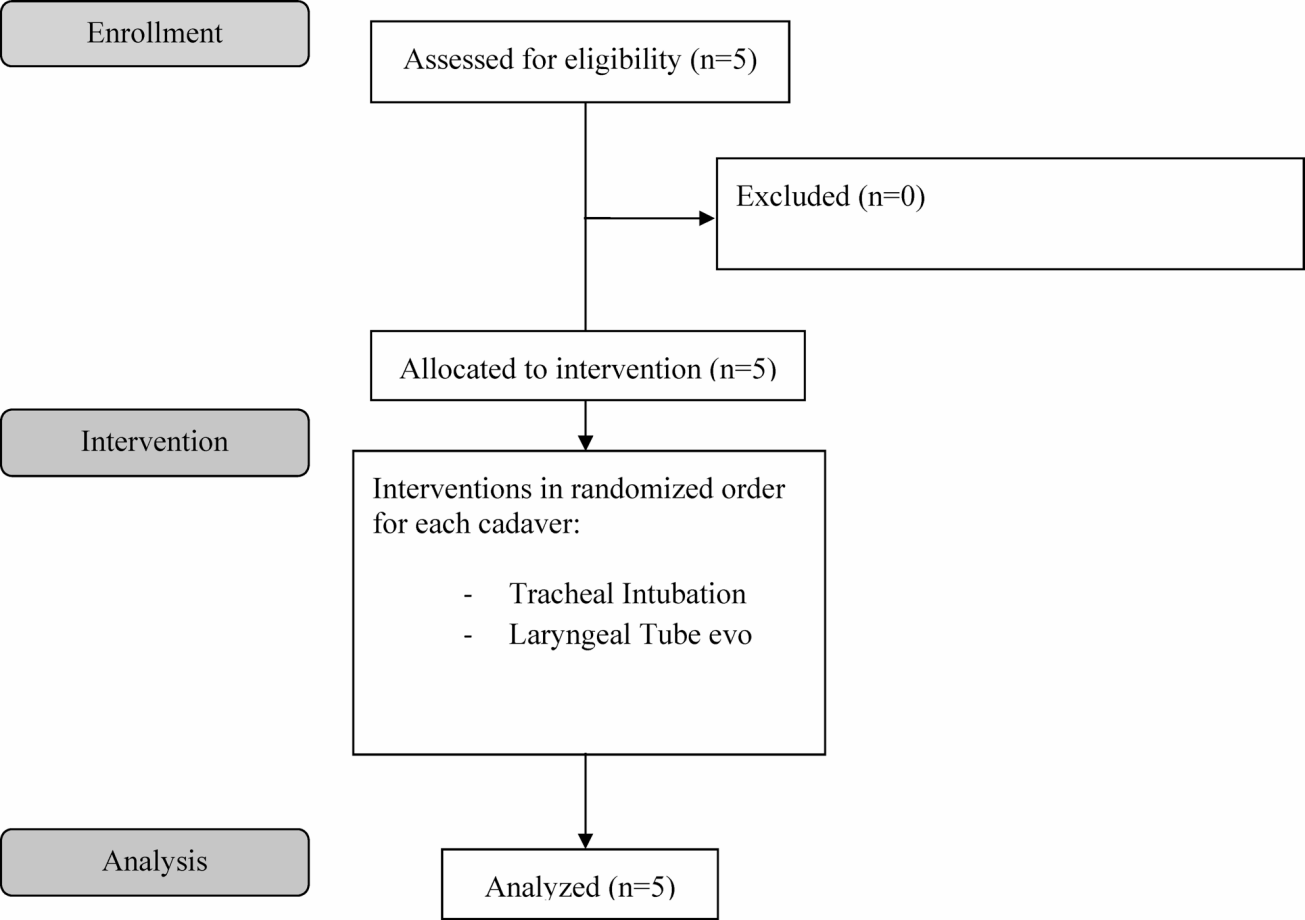
Flow diagram of the study protocol (based on CONSORT 2025)

Five human body donors were included after all the inclusion and exclusion criteria were applied (see Fig. 2).

Table 1 presents the characteristics of the included body donors, and Table 2 provides descriptive statistics of the ventilation parameters according to the different airway devices used.

**Table 1:** Characteristics of the included body donors

No.	Sex (w/m)	Age (years)	Size (cm)	Body weight (kg)	Ideal body weight (kg)	Calculated alveolar dead space (2 ml/kg ideal body weight) (ml)	Ideal tidal volume (ml)	Body mass index (BMI)	Time-to-airway for the LT^®^evo (sec)	Time-to-airway for TI (sec)	Comorbidities
1	m	79	172	65	72	144	504	22	7	10	Prostate carcinoma
2	f	86	152	50	52	104	364	22,5	2	30	Total hip arthroplasty and dementia
3	m	69	180	55	80	160	560	17	12	4	.
4	f	90	162	70	62	124	434	26	2	8	Dementia, heart failure, diabetes mellitus, and liver cirrhosis
5	f	86	165	57	65	130	455	21	13	8	Dementia and pancreatic carcinoma

**Table 2:** Descriptive statistics of the ventilation parameters according to the different airway devices

Variable	Overall (*n* = 455)	Tracheal intubation (*n* = 226)	Laryngeal tube evo (*n* = 229)
Expiratory tidal volume (ml)			
Mean (SD)	194.3 ± 184.8	347.1 ± 144.2	51.3 ± 64.3
Median (Q1; Q3)	106.9 (16.9; 379.8)	379.9 (286.7; 456.4)	19.2 (7.5; 70.3)
Ideal tidal volume (ml)			
Mean (SD)	463.7 ± 66.7	462.5 ± 67.2	464.8 ± 67.0
Median (Q1; Q3)	455 (434; 504)	455 (434; 504)	455 (434; 504)
Ideal – expiratory tidal volume (∆ VT) (ml)			
Mean (SD)	269.4 ± 197.7	115.4 ± 176.0	413.5 ± 60.1
Median (Q1; Q3)	357.7 (49.7; 430.8)	49.5 (4.4; 217.4)	420.6 (371.6; 438.9)
Expiratory tidal volume per kg ideal body weight (ml/kg ideal Bodyweight) (ml)			
Mean (SD)	3.3 ± 3.1	5.8 ± 2.5	0.9 ± 1.1
Median (Q1; Q3)	1.8 (0.3; 6.2)	6.3 (4.7; 7.2)	0.3 (0.1; 1.3)
Leakage Volume (%)			
Mean (SD)	60.3 ± 39.8	32.9 ± 31.2	86.0 ± 28.2
Median (Q1; Q3)	85.3 (24.5; 97.6)	26.9 (5; 46.7)	97.6 (95.1; 99.6)
Inspiratory minute volume (l)			
Mean (SD)	12.9 ± 10.5	6.4 ± 4.8	18.9 ± 10.8
Median (Q1; Q3)	7.5 (3.6; 21.2)	5.3 (3.2; 6.8)	20.6 (8.2; 25.8)
Expiratory minute volume (l)			
Mean (SD)	1.9 ± 1.8	3.4 ± 1.4	0.5 ± 0.6
Median (Q1; Q3)	1.1 (0.2; 3.8)	3.8 (2.9; 4.5)	0.3 (0.1; 0.7)
Maximum Peak Pressure (mbar)			
Mean (SD)	31.7 ± 13.9	41.5 ± 12.3	22.5 ± 7.7
Median (Q1; Q3)	28.1 (22.2; 46.5)	46.5 (36.0; 49.6)	22.42 (21.8; 26.0)
Mean pressure (mbar)			
Mean (SD)	11.1 ± 3.0	12.1 ± 3.0	10.1 ± 2.8
Median (Q1; Q3)	11.5 (10.9; 13.0)	13.0 (11.7; 13.6)	10.9 (10.7; 11.3)
Mean plateau pressure (mbar)			
Mean (SD)	16.6 ± 4.4	16.1 ± 3.9	17.1 ± 4.8
Median (Q1; Q3)	17.7 (16.7; 18.8)	17 (16.0; 17.8)	18.7 (17.6; 19.4)
Legend	SD=standard deviation, Q=quartile		

Table 3: shows the results of the linear mixed regression analyses with random intercepts of various ventilation parameters between the LT^®^evo and TI. The linear mixed regression analyses with random intercepts revealed lower VT_e_ (-295.8 ml; 95%CI: -339.2 to -252.2; *p* < 0.0001), VT_e_/kgIBW (-5.0 ml/kg; 95%CI: -5.7 to -4.2; *p* < 0.0001), MV_e_ (-2.9; 95%CI: -3.4 to -2.5; *p* < 0.0001), P_Peak_ (-19.0mbar; 95%CI: -23.1 to -14.9); *p* < 0.0001), and P_Mean_ (-2 mbar; 95%CI: -3.2 to -0.8; *p* = 0.001) but higher ∆VT (296.0 ml; 95%CI: 252.5 to 339.4; *p* < 0.0001) and V_Leak_ (52.7%; 95%CI: 41.6 to 63.7; *p* < 0.0001) values for the LT^®^evo than for TI. While all the body donors ventilated with TI achieved a VT>2 ml/kgIBW, this was only the case for one (body donor three) using LT^®^evo. Table 4 shows the ventilation parameters that were recorded for the various body donors in detail.

**Table 3:** Results of the linear mixed regression with random intercepts to compare LT^®^evo with tracheal intubation

Covariate Laryngeal tube evo vs. tracheal intubation	Mean difference (regression coefficient)	95% confidence interval	*p*-value
Expiratory tidal volume (ml)	-295.8	-339.2 to -252.2	< 0.0001
Expiratory minute volume (l)	-2.9	-3.4 to -2.5	< 0.0001
Leakage Volume (%)	52.7	41.6 to 63.7	< 0.0001
∆ tidal volume (ml)	296.0	252.5 to 339.4	< 0.0001
Maximum Peak Pressure (mbar)	-19.0	-23.1 to -14.9	< 0.0001
Mean pressure (mbar)	-2	-3.2 to -0.8	0.001
Mean plateau pressure (mbar)	1.0	-0.9 to 2.8	0.3
Respiratory rate (/min)	-0.2	-121 to 0.84	0.72

**Table 4:** Descriptive statistics of the ventilation parameters according to the airway devices for each body donor

Variable	Tracheal intubation	Laryngeal tube evo
**Donor 1**	*n* = 8	*n* = 9
Expiratory tidal volume (ml)	434.9 ± 35.5	78.9 ± 84.0
Ideal tidal volume – expiratory tidal volume (%)	69.1 ± 35.5	425.1 ± 84.0
Expiratory tidal volume (ml) per kg bodyweight	6.7 ± 0.5	1.2 ± 1.3
Expiratory minute volume (l)	4.3 ± 0.4	0.7 ± 0.6
Inspiratory minute volume (l)	5.5 ± 0.6	13.9 ± 7.8
Leakage volume (%)	28.2 ± 2.5	71.0 ± 38.7
Respiratory rate (/minute)	10.0 ± 0.0	9.3 ± 3.0
Mean pressure (mbar)	13.0 ± 0.1	10.2 ± 3.3
Peak pressure (mbar)	47.2 ± 1.1	26.6 ± 12.2
Plateau Pressure (mbar)	17.0 ± 1.4	17.4 ± 5.5
**Donor 2**	*n* = 9	*n* = 9
Expiratory tidal volume (ml)	314.8 ± 107.9	13.8 ± 21.3
Ideal tidal volume – expiratory tidal volume (%)	49.2 ± 107.9	350.2 ± 21.3
Expiratory tidal volume (ml) per kg bodyweight	6.3 ± 2.2	0.3 ± 0.4
Expiratory minute volume (l)	3.1 ± 1.1	0.2 ± 0.3
Inspiratory minute volume (l)	2.2 ± 0.7	21.5 ± 7.6
Leakage volume (%)	4.8 ± 1.2	89.8 ± 28.3
Respiratory rate (/minute)	9.0 ± 2.9	9.1 ± 2.8
Mean pressure (mbar)	12.2 ± 4.0	9.9 ± 3.0
Peak pressure (mbar)	44.3 ± 14.9	20.5 ± 6.5
Plateau Pressure (mbar)	16.0 ± 5.5	16.6 ± 5.2
**Donor 3**	*n* = 9	*n* = 10
Expiratory tidal volume (ml)	159.2 ± 94.5	115.0 ± 74.0
Ideal tidal volume – expiratory tidal volume (%)	400.7 ± 94.5	445.02 ± 74.0
Expiratory tidal volume (ml) per kg bodyweight	2.9 ± 1.7	2.1 ± 1.3
Expiratory minute volume (l)	1.5 ± 0.9	1.1 ± 0.7
Inspiratory minute volume (l)	14.5 ± 3.8	30.2 ± 11.3
Leakage volume (%)	81.8 ± 22.1	85.6 ± 30.2
Respiratory rate (/minute)	9.2 ± 2.5	8.9 ± 3.1
Mean pressure (mbar)	10.5 ± 2.9	10.1 ± 3.6
Peak pressure (mbar)	30.6 ± 9.2	23.5 ± 8.5
Plateau Pressure (mbar)	15.9 ± 4.3	17.1 ± 6.0
**Donor 4**	*n* = 9	*n* = 10
Expiratory tidal volume (ml)	374.8 ± 26.8	26.5 ± 21.6
Ideal tidal volume – expiratory tidal volume (%)	59.2 ± 26.8	407.5 ± 21.6
Expiratory tidal volume (ml) per kg bodyweight	5.3 ± 0.4	0.4 ± 0.3
Expiratory minute volume (l)	3.8 ± 0.2	0.3 ± 0.2
Inspiratory minute volume (l)	6.6 ± 0.5	20.6 ± 6.1
Leakage volume (%)	45.2 ± 4.6	92.3 ± 20.0
Respiratory rate (/minute)	9.8 ± 0.7	9.4 ± 2.7
Mean pressure (mbar)	12.4 ± 0.8	10.1 ± 2.1
Peak pressure (mbar)	38.3 ± 2.4	20.2 ± 4.4
Plateau Pressure (mbar)	15.9 ± 1.3	16.6 ± 3.6
**Donor 5**	*n* = 9	*n* = 9
Expiratory tidal volume (ml)	461.5 ± 158.8	18.1 ± 7.0
Ideal tidal volume – expiratory tidal volume (%)	-6.5 ± 158.8	436.9 ± 7.0
Expiratory tidal volume (ml) per kg bodyweight	8.1 ± 2.8	0.3 ± 0.1
Expiratory minute volume (l)	4.6 ± 1.6	0.2 ± 0.1
Inspiratory minute volume (l)	3.1 ± 1.0	6.8 ± 2.0
Leakage volume (%)	4.1 ± 1.5	90.6 ± 20.8
Respiratory rate (/minute)	9.0 ± 3.1	9.3 ± 2.2
Mean pressure (mbar)	12.5 ± 4.2	10.2 ± 2.4
Peak pressure (mbar)	47.9 ± 16.4	21.7 ± 4.2
Plateau Pressure (mbar)	15.7 ± 5.3	17.6 ± 4.6

## Discussion

### Main findings

This study investigated the influence of the new LT^®^evo vs. TI on the VT_e_ of human body donors during intra-arrest-ventilation using biphasic-positive-airway-pressure-ventilation during mechanical chest compressions. The comparison of the LT^®^evo and TI revealed lower VT_e_, VT_e_/kgIBW, MV_e_, and P_Mean_ but higher ∆VT and V_Leak_ values for LT^®^evo.

### Airway management in out-of-hospital cardiac arrest

The optimal airway management using TI vs. SGA devices in advanced cardiac life support has been controversially discussed for decades. While TI is often reserved for experienced airway managers because of its complexity, SGA devices are frequently used by nonmedical emergency personnel because their use is easy to learn, they have a high first-pass success rate, and they can be placed quickly without interrupting chest compressions [7, 15]. While the guidelines of the American Heart Association recommend the use of SGA devices or TI depending on the user’s level of training [15], the European Resuscitation Council recommends the preferred use of an i^−^gel^®^ laryngeal mask over a laryngeal tube for the first time because of varying success, complication, and survival rates [7, 16–18]. The LT^®^evo is a recent advancement of LTS-D, but there is currently a lack of experience regarding its use and its effects on intra-arrest ventilation. In the present study, the LT^®^evo could be inserted quickly, which suggests that its use in out-of-hospital resuscitation is entirely feasible.

### Intra-arrest ventilation

The use of intra-arrest ventilation has been recommended in guidelines for decades; however, unlike airway management, defibrillation, and other procedures, there is significantly less evidence regarding its optimal implementation [19–22].

Although oxygenation and ventilation during advanced cardiovascular life support are often inadequate and associated with poor outcomes, studies evaluating optimal initial airway management in patients with out-of-hospital cardiac arrest often fail to consider the importance of the interaction between the airway device inserted and the intra-arrest ventilation performed [1–6, 22]. However, the results of the present study suggest that the various airway devices do indeed influence the target parameters of the selected intra-arrest ventilation.

After the airway is secured, asynchronous ventilation is recommended in the guidelines; continuous chest compressions should be continued with a respiratory rate of 10/min or every six seconds, traditionally using bag-valve-mask ventilation or, in the case of mechanical ventilation, using volume-controlled-ventilation or pressure-regulated modes [7, 15]. In addition to volume-controlled-ventilation, prehospital emergency ventilators are now often available, enabling the use of other ventilation modes potentially suitable for intra-arrest ventilation, such as biphasic-positive-airway-pressure-ventilation [21]. However, these have become significantly less common, particularly with the use of SGA, and the new LT^®^evo has not yet been evaluated [8, 9]. A randomized study of out-of-hospital cardiac arrest patients revealed significantly higher VT for biphasic-positive-airway-pressure-ventilation than for continuous positive airway pressure after TI, which was not the case vs. volume-controlled-ventilation [9]. However, unlike in a retrospective registry study, no differences in the return of spontaneous circulation rate were observed for biphasic-positive-airway-pressure-ventilation [8, 23]. The present study is the first to evaluate TI versus the new LT^®^evo in the context of intra-arrest ventilation using biphasic-positive-airway-pressure-ventilation in human body donors. Higher VT_e_ and VT_e_/kgIBW values were observed for TI because of significantly fewer leaks. There are several possible reasons for this outcome: (1) While SGA can undergo dynamic position changes with (sub)luxation because of head or neck movements during resuscitation caused by chest compressions, the risk is significantly lower for well-fixed tracheal tubes. (2) Although the cuffs were blocked at the same cuff pressure of 40 cmH2O for better comparability, the manufacturer specifies that blocking ≤ 60 cmH2O is possible for the LT^®^evo. In the context of intra-arrest ventilation via the LT^®^evo, blocking up to 60 cmH2O could therefore be useful to reduce leakage. (3) Thiele fixation may have led to a change in the anatomy or tissue texture of the upper airways, influencing the results in comparison to those for unfixed patients requiring resuscitation. (4) In accordance with the guidelines, a PEEP of five mbar was selected for biphasic-positive-airway-pressure-ventilation in this study [7]. While this has minimal effects on the TI position when the cuff is blocked, with SGA such as the LT^®^evo, it is theoretically possible that PEEP could lead to (partial) dislocation of the SGA, particularly in a dynamic resuscitation situation, thereby increasing leakage.

The principal objective of intra-arrest ventilation is to achieve the best possible oxygenation and decarboxylation, thereby limiting the metabolic acidosis frequently observed during cardiac arrest. Chest-compression-synchronized ventilation, developed specifically for CPR [20], has been shown in animal models to improve blood gas parameters even when delivered tidal volumes approximate anatomical dead space. In contrast, biphasic positive airway pressure ventilation depends on tidal volumes that exceed anatomical dead space for achieve effective alveolar gas exchange. In this study, the higher VT_e_ and VT_e_/kgIBW observed with TI, together with increased ΔVT and V_Leak_ associated with LT^®^evo, are consistent with prior reports and may account for the less favourable blood gas values previously described with SGA use compared with TI during mechanical cardiopulmonary resuscitation (mCPR) [20, 21, 24, 25]. These findings suggest that ventilation via SGA during mCPR may frequently result in delivered tidal volumes that approach or fall below anatomical dead space. Notably, use of the LT^®^evo resulted in tidal volumes approximating anatomical dead space in only one body donor, indicating that asynchronous intra-arrest ventilation with the LT^®^evo during mCPR may frequently provide ventilation at or even below dead-space levels, raising concern that effective alveolar ventilation may not be reliably achieved (see Table 4). Although current guidelines recommend ensuring the effectiveness of mechanical ventilation and switching to manual ventilation when mechanical ventilation is ineffective, the available data suggest that use of LT^®^evo may favour synchronized ventilation at a ratio of 30:2 rather than continuous ventilation during mCPR, especially given the limited availability of reliable prehospital monitoring of intra-arrest ventilation [7].

### Limitations

Our study has several limitations that should be considered when the findings are interpreted. The limited sample size may limit the generalizability of the results. Nevertheless, the available data yielded statistically significant results that indicate sufficient statistical power. The study was conducted under highly controlled laboratory conditions, which only partially reflect the real-life conditions involved in a genuine prehospital emergency response. The limitation of the duration of resuscitation to four minutes allows only limited validity in comparison with that of resuscitation measures, which are sometimes necessary for much longer durations. Owing to the limited availability of Thiel-embalmed body donors, chest-compression-synchronised-ventilation and volume-controlled-ventilation were examined on the same body donors in addition to biphasic-positive-airway-pressure-ventilation. Each ventilation mode was evaluated with each airway device for a period of four minutes, resulting in a total ventilation time under chest compressions of 48 min for each body donor, an approach that may have had a decisive impact on the integrity and viscoelastic properties of the tissue and therefore potentially influenced the parameters measured. In the present study, both the ventilation patterns used and the airway devices used were randomized to ensure that the possible effects of continuous resuscitation time on the body donors were evenly distributed. However, continuous resuscitation is frequently prolonged in the prehospital setting. It is possible that the predefined limitation of the cuff pressure to 40 cmH2O for comparability between devices may have contributed to leakage. A limitation of up to 60 cmH2O is possible, particularly for SGA, which could contribute to a reduction in leakage with the LT^®^evo. Furthermore, the use of different airway devices may have influenced the ventilation conditions for subsequent airway devices, e.g., because of alveolar collapse. However, the order of the airway devices was randomized, and after each new insertion, the lungs were recruited again. Despite these limitations, this study provides valuable insights into the effects of different airway devices during intra-arrest ventilation in a randomized and controlled human body donor model. Furthermore, the present study is the first to evaluate the effects of intra-arrest ventilation with biphasic-positive-airway-pressure-ventilation under mCPR using the new LT^®^evo, thereby providing insights into this novel airway device.

## Conclusions

Compared with TI, asynchronous intra-arrest ventilation via biphasic-positive-airway-pressure-ventilation under continuous mCPR using the LT^®^evo resulted in lower VT_e_, VT_e_/kgIBW, P_Peak_, P_Mean_, and P_Plat_ values but higher V_Leak_ and ΔVT values. Whereas all the tracheally intubated body donors achieved VT exceeding anatomical dead space, this threshold was reached in only one body donor when LT^®^evo was used. These findings suggests that LT^®^evo may be ill suited for asynchronous intra-arrest ventilation, as effective ventilation cannot be ensured under these conditions. When airway management is performed with the LT^®^evo, a synchronized ventilation strategy using a 30:2 compression-to-ventilation ratio may therefore represent a safer alternative. Future studies should evaluate the influence of different airway devices in mCPR on intra-arrest ventilation in terms of individualized CPR.

## Supplementary Information

Below is the link to the electronic supplementary material.

Supplementary Material 1

## Data Availability

The datasets used and/or analysed during the current study are available from the corresponding author upon reasonable request.

## Abbreviations

LT®evo: Laryngeal tube evo
TI: Tracheal tube/tracheal intubation
CI: Confidence interval
(m)CPR: (mechanical) cardiopulmonary resuscitation
∆VT: Difference between VT_Ideal_ and VT_e_ (VT_Ideal_–VT_e_)
IBW: Ideal body weight
LTSD: Laryngeal tube
PEEP: Positive end-expiratory pressure
P_Insp_: Inspiratory pressure
P_Mean_: Mean pressure
P_Peak_: Peak pressure
P_Plat_: Plateau pressure
SGA: Supraglottic airway device
V_Leak_: Leakage volume
VT: Tidal volume
VT_e_: Expiratory tidal volume
VT_e_/kgIBW: VT_e_ per kilogram of ideal body weight
VT_Ideal_: Ideal tidal volume
MV_i_: Inspiratory minute volume
MV_e_: Expiratory minute volume
