# Supplementary material for: Comparison of laryngeal tube evo with tracheal intubation during intra-arrest-ventilation using biphasic positive pressure ventilation - a prospective randomized controlled body donor study
Source: Crit Care. 2026 Mar 28;30:166. doi: 10.1186/s13054-026-05902-4 (PMC13064349; doi:10.1186/s13054-026-05902-4)
Supplement: Supplementary file 1 — Supplementary Material 1 [file 13054_2026_5902_MOESM1_ESM.docx]

**Additional material 1 – Table of measured ventilation parameters and calculation methods**

| **Parameters** | **Description** | **Calculation** |
| --- | --- | --- |
| VT_e_ (ml) | Expiratory tidal volume, approximation for actual tidal volume | Sum of expiratory flow values (averaged over five breaths):  Times for inspiration and expiration sought on pressure curve (pressure approx. zero), transfer to flow curve, integral over expiration. |
| ∆VT (ml) | Parameters for visualizing effective ventilation | ∆VT = VT_ideal_–VT_e_ |
| VT_ideal_ (ml) | Calculated tidal volume based on the ideal body weight (IBW) | Calculated by the mechanical ventilator:  IBW female (kg) = 45 + 2·3 x (height in cm/2·54−60)  IBW male (kg) = 50 + 2·3 x (height in cm/2·54−60)  multiplicated with 6 ml |
| MV_e_ (l/min) | Expiratory volume per minute | Minute average of the expiratory tidal volume measured by the device.  MVe=VT×F, averaged over five breaths |
| MV_i_ (l/min) | Inspiratory volume per minute | Minute average of the inspiratory tidal volume measured by the device.  MVe=VT×F, averaged over five breaths |
| V_leak_ (%) | Relative leakage volume | 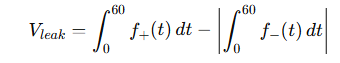Integral of the flows over one minute (positive flows - negative flows) divided by the inspiratory minute volume. |
| P_peak_ (mbar) | Peak pressure | Mean value of the maximum airway pressure (peak pressure) per respiratory cycle over one minute. |
| P_mean_ (mbar) | Mean pressure | Average value of the pressure over one minute. All pressure values measured during a breath were totaled and divided by the number of time intervals in which the measurement was taken. |
| P_plat_ (mbar) | Plateau pressure | Pressure value measured by the device in the last 30 ms of inspiration, averaged over five breaths. |
